# Pozelimab for CHAPLE disease: results from in-trial interviews and clinical outcome assessments

**DOI:** 10.1186/s13023-024-03277-9

**Published:** 2024-08-08

**Authors:** Leighann Litcher-Kelly, Ahmet Ozen, Sarah Ollis, Hagit Baris Feldman, Andrew Yaworsky, Paolo Medrano, Voranush Chongsrisawa, Taylor Brackin, Lorah Perlee, Marisa Walker, Sharanya Pradeep, Michael J. Lenardo, Olivier A. Harari, Jessica J. Jalbert

**Affiliations:** 1 Adelphi Values, Boston, MA USA; 2 Marmara University, Istanbul, Türkiye; 3 Tel Aviv Sourasky Medical Center and Sackler Faculty of Medicine, Tel Aviv University, Tel Aviv, Israel; 4 Department of Pediatrics, Faculty of Medicine, Chulalongkorn University, King Chulalongkorn Memorial Hospital, Thai Red Cross Society, Patumwan, Bangkok, Thailand; 5 Regeneron Pharmaceuticals, Inc., 777 Old Saw Mill River Road, Tarrytown, NY 10591 USA; 6 Molecular Development of the Immune System Section, Laboratory of Immune System Biology, Laboratory of Clinical Immunology and Microbiology, and Clinical Genomics Program, National Institute of Allergy and Infectious Diseases, National Institutes of Health, Bethesda, MD USA

**Keywords:** (3–10): CHAPLE disease, COA, Open-label trial, Pozelimab, Within-trial interviews

## Abstract

**Background:**

CD55 deficiency with hyper-activation of complement, angiopathic thrombosis, and protein-losing enteropathy (CHAPLE) disease is ultra-rare (< 100 children or young adults worldwide) and potentially fatal. The study used mixed-methods approaches to assess how pozelimab impacts the signs and symptoms of CHAPLE disease from the patient perspective by combining within-trial interviews and clinical outcome assessments (COAs) (ClinicalTrials.gov, NCT04209634).

**Methods:**

Interviews conducted with patients/caregivers at screening and week 24 assessed the signs and symptoms of CHAPLE disease, including those which were most bothersome, and evaluated the change. Patients/caregivers and clinicians completed the COAs; interview data contextualized the meaningfulness of change.

**Results:**

Ten patients (aged 3–19 years) were enrolled; caregivers contributed to nine interviews. Abdominal pain, diarrhea, facial and peripheral edema, nausea, and vomiting are the core signs and symptoms of CHAPLE disease (≥ 90% patients experienced pre-treatment); the most bothersome signs and symptoms were abdominal pain (*n* = 9) and facial edema (*n* = 1). All core signs and symptoms were reported as resolved at week 24 interviews. Severity on global assessments changed from “mild” to “very severe” at baseline to “no signs or symptoms” at week 24. Interview results were generally consistent with sign- or symptom-specific COA scores.

**Conclusions:**

Patients with CHAPLE disease treated with pozelimab for 24 weeks experienced complete resolution of core signs and symptoms. Mixed-methods approaches can contextualize the patient experience (how patients feel and function) in rare disease trials.

**Trial registration:**

Clinicaltrials.gov, NCT04209634, registered December 24, 2019, https://classic.clinicaltrials.gov/ct2/show/NCT04209634.

**Supplementary Information:**

The online version contains supplementary material available at 10.1186/s13023-024-03277-9.

## Introduction

CD55 deficiency with hyper-activation of complement, angiopathic thrombosis, and protein-losing enteropathy (CHAPLE) disease is an ultra-rare, potentially fatal condition caused by mutations in the *CD55* gene. These mutations prevent normal cell surface expression of the CD55 protein, which regulates the complement system, by accelerating the inactivation of C3 and C5 convertases [1]. Loss of CD55 causes primary intestinal lymphangiectasia and protein-losing enteropathy, which can result in severe abdominal pain, chronic/recurrent diarrhea, vomiting, and edema that can require frequent hospitalizations and medical interventions [1]. Patients experience malabsorption, resulting in impaired growth, anemia, and vitamin and nutrient deficiencies [1]. This newly described disease is associated with high mortality [2, 3]. Estimates suggest there are fewer than 100 patients worldwide [4] and, prior to the August 2023 approval of pozelimab (ClinicalTrials.gov: NCT04209634) [5] by the US Food and Drug Administration (FDA), management for the condition was supportive and used a similar therapeutic approach to that for acquired or familial forms of protein-losing enteropathy, e.g. corticosteroids, biologics, immunomodulators, intravenous (IV) or subcutaneous (SC) immunoglobulin, IV albumin, micronutrients, and restricted diets [1, 3, 6]. Additionally, off-label IV administration of eculizumab has been shown to ameliorate CHAPLE disease [2, 7]. Pozelimab is an SC-administered fully human monoclonal immunoglobulin G4P antibody directed against the terminal complement protein C5, a key protein for activation of the terminal pathway of the complement system. The SC administration of pozelimab may be less burdensome and costly to patients compared to treatments requiring IV administration [8, 9].

The FDA has advanced patient-focused drug development guidance documents and dual-methodological approaches (i.e. “mixed methods” using qualitative and quantitative information) as a means to incorporate the patient perspective into clinical trials for rare diseases [10, 11]. Given the rarity of the condition and the trial’s small sample size, and the potential heterogeneity of the symptomology, within-trial interviews and clinical outcome assessment (COA) questionnaires were used to evaluate the signs and symptoms of CHAPLE disease, including each participant’s most bothersome sign/symptom (MBS) [12], from the perspective of the patient before and during treatment with pozelimab. Here, we report on the treatment benefit of pozelimab on the signs and symptoms of CHAPLE disease, an ultra-rare condition, using a novel mixed-methods approach combining within-trial interviews and COA data to better contextualize the patients’ experience of the medication’s effects.

## Methods

### Trial design

NCT04209634 is an ongoing, open-label, single-arm, multicenter, phase 2/3 study evaluating the efficacy and safety of pozelimab in pediatric and adult patients with CHAPLE disease. Methods and results of the trial, including details on the study design, inclusion/exclusion criteria, and the efficacy and safety results, are described elsewhere [13, 14], while the research described herein focuses on the patient experience of signs and symptoms of the condition. Patients aged ≥ 1 year with a clinical and genetically confirmed CHAPLE disease diagnosis and active or inactive/controlled disease were eligible. At baseline (day 1), patients received a single IV loading dose of pozelimab 30 mg/kg, followed by SC dosing based on body weight once weekly over the treatment period. During clinical trial planning, potential core sign and symptom concepts were identified by reviewing the published literature and through discussions with treating clinicians. Six signs and symptoms (abdominal pain, facial edema/swelling, peripheral edema/swelling, nausea, diarrhea, and vomiting) were selected for the COA measurement strategy, as key features of the disease experience which were expected to improve with treatment [15]. Given the potential for heterogeneity in the experience of disease between patients and the possibility that patients could select a sign/symptom that could not ameliorate with treatment (e.g., speech disability following a stroke), each participant selected the sign/symptom that was most bothersome before starting pozelimab from a list of potential core signs and symptoms identified through clinician interviews [12, 16, 17].

### Interview methods

Two 60-minute interviews were conducted at screening and week 24 with all trial participants and/or their caregivers. Screening interviews were conducted prior to initiating pozelimab and were used to confirm the core signs and symptoms as well as to document each patient’s MBS; week 24 interviews were conducted at the week 24 clinical trial visit to understand the impact of treatment with pozelimab on the patient’s experience of their disease. Patients ≥ 8 years of age were the primary respondents of the interview, with input from their caregiver as appropriate; for patients < 8 years old, or those with cognitive impairments, the caregiver was the primary or sole respondent of the interview, although the patient was also invited to contribute.

Each interview was conducted by a trained interviewer in person or via telephone (an option for the week 24 interviews due to the COVID-19 pandemic) in the native language of the participant. Except for one participant whose interviews were conducted by a research nurse at the site, all other interviews were conducted by persons independent from the clinical site staff. All interviewers were trained in qualitative interview methodology. The interviews were audio-recorded, transcribed, anonymized, and translated into US English by an independent transcription company. Transcripts were anonymized by removing any potentially identifying information (e.g. names of people and places) from the transcripts.

### Clinical outcome assessments

Given the inherent constraints of studying an ultra-rare disease group, COAs included in the clinical trial were selected with the goal of following best measurement practices summarized in regulatory guidance documents [10]. Depending on the age of the patient, the primary respondent of the COAs was either the patient themselves (if aged ≥ 12 years) or the caregiver (if aged < 12 years).

Global assessments of symptom/disease severity and change were completed by patients or caregivers and clinicians. Specifically, the patient/caregiver global impression of severity (GIS) questions assessed overall sign and symptom severity using a five-point response scale ranging from absent (0) to very severe (4) [18, 19]. A similar approach was used for the patient/caregiver global impression of change (GIC) questions, except that the change in the disease severity was assessed on a seven-point response scale ranging from much worse (–3) to no change (0) to much better (3). Clinicians also rated global disease severity and change using the same five-point and seven-point scales for clinician GIS and GIC, respectively.

Several COAs were administered to assess potential core signs and symptoms: the Pediatric Quality of Life Inventory (PedsQL™) gastrointestinal symptom scales (pain and hurt, diarrhea, and nausea/vomiting subscales) [20], facial edema physician assessment, and peripheral edema physician assessment. These are summarized in Table 1 and Additional file 1.

**Table 1:** Sign- and symptom-specific COAs by core signs and symptoms

Core sign/symptom assessed	Sign/symptom-specific COA	Score range
Abdominal pain	PedsQL™ GI symptoms stomach pain and hurt subscale	0-100; higher scores = less frequency of problem
Diarrhea	PedsQL™ GI symptoms diarrhea subscale	0-100; higher scores = less frequency of problem
Facial edema/swelling	Physician assessment of facial edema	0–5; higher scores = worse edema
Peripheral edema/swelling	Physician assessment of peripheral edema	0–5; higher scores = worse edema
Nausea and vomiting	PedsQL™ GI symptoms nausea and vomiting subscale	0-100; higher scores = less frequency of problem

COA, clinical outcomes assessment; GI, gastrointestinal; PedsQL™, Pediatric Quality of Life

### Analysis

Interview data elicited from patients and caregivers from the screening and week 24 interviews were coded for each patient, with specific attention to any changes in the signs and symptoms identified as key during trial planning, along with the MBS that was reported at the screening interview. Coding and analysis were performed on the translated transcripts, using qualitative data analytic methods and following a pre-specified qualitative data analysis plan. Interview data were pooled across participants and were analyzed for concept frequency and concept descriptions.

To evaluate overall improvement by week 24 for each participant, both information from the week 24 interviews about signs/symptoms and scores on the global assessments of severity and change (reported by patient/caregiver and clinician) are presented per patient.

Additionally, changes in COA scores are reported alongside example interview quotes from participants describing their experience of the sign or symptom and how it changed during treatment. Specifically, to understand the response to pozelimab treatment and whether it was meaningful to patients, key quotes from the screening and week 24 interviews were used to contextualize COA change scores in specific concepts and the overall disease experience gathered during the trial, and these were compared to scores on the GIS. Change on the COAs from baseline to week 24 (mean, median, minimum, and maximum) was evaluated at the patient level to assess whether the participant’s sign or symptom improved, did not change, or worsened by week 24.

Finally, for each patient’s MBS, the associated sign- or symptom-specific COA scores and patient- or caregiver-reported GIS/GIC scores are summarized, along with patient/caregiver quotes about the MBS at screening and week 24 interviews as well as the meaningfulness of the change.

## Results

### Patient characteristics

Ten patients were enrolled in the clinical trial at three sites in Türkiye (*n* = 7), Thailand (*n* = 2), and the USA (*n* = 1). The mean age of patients was 9.3 years (standard deviation = 4.9), and more than half were female (*n* = 6; 60.0%). Additional demographic and health information, as well as trial efficacy and safety results, are presented elsewhere [14].

One patient completed both the screening and week 24 interviews independently, while the remaining interviews were conducted either as dyads (*n* = 5 at screening and *n* = 6 at week 24) or with the caregiver only (*n* = 4 at screening and *n* = 3 at week 24). Similarly, the COAs were completed by caregivers for most patients (*n* = 8). Following pozelimab treatment, all 10 patients met the composite primary endpoint of albumin normalization and improvement (or no worsening) in clinical signs and symptoms at week 24 (reported elsewhere). Table 2 summarizes the demographic information of the participants, as well as the respondent information specific to the interviews and COAs.

**Table 2:** Site-reported patient demographic information (*n* = 10)

Characteristic	Total (*N* = 10)
Age, years	
Median (range)	8.5 (3–19)
Mean (standard deviation)	9.3 (4.9)
Age groups, years, *n* (%)	
≤ 7	4 (40.0)
≥ 8–<12	4 (40.0)
≥ 12–<18	1 (10.0)
≥ 18	1 (10.0)
Sex, *n* (%)	
Female	6 (60.0)
Male	4 (40.0)
Interview participants (screening/week 24),^a ^*n* (%)	
Patient only	1 (10.0)/1 (10.0)
Patient/caregiver dyad	5 (50.0)/6 (60.0)
Caregiver only	4 (40.0)/3 (30.0)
Respondent for COAs,^b ^*n* (%)	
Patient	2 (20.0)
Caregiver	8 (80.0)
Country of origin (native language),^c ^*n* (%)	
Bolivia (Spanish)	1 (10.0)
Syria (Arabic)	2 (20.0)
Thailand (Thai)	2 (20.0)
Türkiye (Turkish)	5 (50.0)

COA, clinical outcome assessment

^a^Generally, patients aged ≥ 8 years with or without their caregivers were the primary respondent for interviews; for patients aged < 8 years, caregivers were the primary respondents

^b^Patients ≥ 12 years old completed COAs; caregivers of patients < 12 years old completed COAs.

^c^While most patients (*n* = 7, 70.0%) received treatment in their native country, the patients from Bolivia (*n* = 1, 10.0%) and Syria (*n* = 2, 20.0%) were treated in the USA and Türkiye, respectively

### Screening interview results

Prior to initiating treatment, nine patients (90.0%) reported experiencing all of the following signs and symptoms: abdominal pain, diarrhea, facial edema/swelling, nausea, peripheral edema/swelling, and vomiting; one patient (10.0%) reported experiencing all these except for nausea. Other signs and symptoms were reported by ≤ 3 patients. Abdominal pain, diarrhea, facial edema/swelling, nausea, peripheral edema/swelling, and vomiting were the signs and symptoms identified by the literature and clinicians at the trial planning stage as being central to the patient’s disease experience. Therefore, the results of the interviews confirmed that the six signs and symptoms selected for the COA measurement strategy were central to the patient’s disease experience, and were the core signs/symptoms of CHAPLE disease [15].

Abdominal pain was reported to be the MBS for nine out of 10 patients (90.0%); for the remaining patient (*n* = 1, 10.0%), facial edema was the MBS. Furthermore, before starting treatment most patients (*n* = 7, 70.0%) reported that abdominal pain was the most important symptom that they would like to see improved with treatment.

## Week 24 interview results

All patients experienced a complete resolution of core signs and symptoms at the week 24 interview (Table 3). Moreover, 19 signs and symptoms were discussed in the context of change (including the six core signs and symptoms) [15], and nine participants (90.0%) had improvement in all signs and symptoms; the remaining patient (*n* = 1, 10.0%) experienced an improvement in all signs and symptoms except for one, the inability to gain weight, which did not change following treatment. Of note, no patients reported worsening of any signs and symptoms.

**Table 3:** Example quotes of overall improvement over the clinical trial

Complete resolution participant quotes (*N* = 10)	
**Patient #1** CAREGIVER: … after taking this drug, the symptoms did not show up any more.… we can live normally. **Patient #2:** CAREGIVER: Since taking the medication, since joining the study, he hasn’t had any symptoms at all… Before taking the medication, everything seemed horrible during kindergarten and in terms of physical and social matters. But now, it’s like we can change the situation, something like that. We feel better. **Patient #3:** CAREGIVER: He did not have any abdominal pain or vomiting…. There has been a very nice change. Happy. In other words, he does not get sick. We are glad. I mean, he is like the other children, I am very happy. My child gets better day by day and this is very good.^a^ **Patient #4:** CAREGIVER: Swelling disappeared; nausea, abdominal pain, diarrhea: all of these disappeared. These were the complaints of her/his disease on their own, but these disappeared… I was very happy; I cannot explain that feeling. You cannot express it with words. That is something in that moment. You live with the fear of recurrence, but it does not happen and the fact that it does not happen is a better thing. **Patient #5**: PATIENT: I used to have a headache, stomachache, nausea – all of these complaints in the past. Now I don’t have any of these. **Patient #6:** CAREGIVER: All the symptoms disappeared… **Patient #7:** CAREGIVER: All stopped after taking the medication… Everything was bad 6 months ago, and now everything is good. **Patient #8:** PATIENT: All of them, all symptoms disappeared. I sometimes had mild edema, but in a manner to occur in a normal person; in a way everybody can have. All symptoms disappeared in terms of my disease: abdominal pain, nausea, well, vomiting, diarrhea, etc.; particularly the edema. All of these disappeared… **Patient #9:** CAREGIVER: The swelling in her eyes disappeared. Her abdominal pain, fever, and diarrhea symptoms were resolved completely. I mean, she had a lot of improvement. The swelling started to disappear right away after the first dose was given; however, diarrhea and abdominal pain were resolved almost before the swelling. As she had water in her body, that was resolved slightly later but diarrhea and abdominal pain were resolved right away after she got the first dose.… **Patient #10:** PATIENT: Now I feel normal; I am no longer in pain; I can do whatever I want….	

^**a**^Caregiver reported complete resolution of all core signs and symptoms; however, the patient reported very mild nausea due to overeating

### Overall improvement in signs and symptoms/disease activity at week 24

The interview findings align with the scores on the global assessments (Table 4). Specifically, all patients (*N* = 10, 100.0%) had no signs or symptoms at week 24 (as reported by both patients or caregivers and their clinicians), and nearly all patients indicated that their change from baseline for overall disease severity on the GIC was “much better” (*n* = 9, 90.0%). One patient (10.0%) had a GIC (reported by the caregiver and the clinician) of “a little better,” but it should be noted that the caregiver and clinician reported the patient’s baseline severity as mild. Of note, for a small number of patients there were some slight discrepancies at baseline between patient/caregiver assessments and clinician assessments. However, at Week 24, participant and clinician global ratings post-treatment were aligned for all patients; all patients or caregivers and clinicians reported a positive impact of treatment on disease activity.

**Table 4:** GIS and GIC for overall CHAPLE disease signs and symptoms

Participant #	Respondents	Screening (pre-treatment) global assessment of severity		Week 24 (post-treatment) global assessment of severity		Week 24 global assessment of change	
		Patient/ caregiver GIS score	Clinician GIS score	Patient/ caregiver GIS score	Clinician GIS score	Patient/ caregiver GIC score	Clinician GIC score
#1	Caregiver and clinician	3 (severe)	1 (mild)	0 (absent [no symptoms])	0 (absent [no signs or symptoms])	3 (much better)	3 (much better)
#2	Caregiver and clinician	4 (very severe)	3 (severe)	0 (absent [no symptoms])	0 (absent [no signs or symptoms])	3 (much better)	3 (much better)
#3	Caregiver and clinician	1 (mild)	1 (mild)	0 (absent [no symptoms])	0 (absent [no signs or symptoms])	1 (a little better)	1 (a little better)
#4	Caregiver and clinician	1 (mild)	1 (mild)	0 (absent [no symptoms])	0 (absent [no signs or symptoms])	3 (much better)	3 (much better)
#5	Caregiver and clinician	2 (moderate)	2 (moderate)	0 (absent [no symptoms])	0 (absent [no signs or symptoms])	3 (much better)	3 (much better)
#6	Caregiver and clinician	1 (mild)	2 (moderate)	0 (absent [no symptoms])	0 (absent [no signs or symptoms])	3 (much better)	3 (much better)
#7	Caregiver and clinician	1 (mild)	2 (moderate)	0 (absent [no symptoms])	0 (absent [no signs or symptoms])	3 (much better)	3 (much better)
#8	Patient and clinician	2 (moderate)	3 (severe)	0 (absent [no symptoms])	0 (absent [no signs or symptoms])	3 (much better)	3 (much better)
#9	Caregiver and clinician	1 (mild)	2 (moderate)	0 (absent [no symptoms])	0 (absent [no signs or symptoms])	3 (much better)	3 (much better)
#10	Patient and clinician	3 (severe)	2 (moderate)	0 (absent [no symptoms])	0 (absent [no signs or symptoms])	3 (much better)	3 (much better)

CHAPLE, CD55 deficiency with hyper-activation of complement, angiopathic thrombosis, and protein losing enteropathy; GIC, global impression of change; GIS, global impression of severity

GIS range of scores is based on a five-point Likert scale (0–4), where a score of 0 represents “no symptoms” and 4 represents “very severe.” Higher scores indicate worse symptoms. GIC scores are based on the following response options: −3, much worse; −2, moderately worse; –1, a little worse; 0, no change; 1, a little better; 2, moderately better; and 3, much better. Higher scores indicate more improvement

Table 5 presents the median change in scores on the COAs summarized across participants for each core sign or symptom of CHAPLE disease, along with example quotes. For abdominal pain, responses to the PedsQL™ Pain and Hurt subscale (i.e. abdominal pain) from baseline to week 24 indicated a median improvement of 43.8 points (*N* = 10; range, 0.0–91.7). For facial edema, the physician assessments from baseline to week 24 indicated a median improvement of one point (*n* = 10; range, 0–3). Eight patients (80.0%) had improved scores on the facial edema physician assessment by week 24; the two patients (20.0%) who did not have a change in their scores had baseline scores of “mild edema” and “no edema.” For the COA assessments for the other core signs and symptoms (diarrhea, peripheral edema/swelling, nausea, and vomiting), most patients (*n* ≥ 8) had scores that indicated improvement and/or resolution of the symptom from baseline at week 24. Those who did not report improvement either were not experiencing the sign or symptom at baseline or had reported the severity at baseline to be mild.

**Table 5:** Clinical outcome assessment response from baseline to week 24, and accompanying example quotes from the week 24 interviews

Median (range) score at baseline *n* (%)	Median (range) score at week 24 *n* (%)	Median (range) change from baseline to week 24 *n* (%)	Response *n* (%)
Abdominal pain: PedsQL™ Pain and Hurt subscale^a^			
56.25 (0.0–100.0) 10 (100.0)	100 (91.7–100.0) 10 (100.0)	43.75 (0.0–91.7) 10 (100.0)	Improve: 9 (90.0) No change: 1 (10.0)
Quote: CAREGIVER: Since taking the medication, since joining the study, he hasn’t had any symptoms at all…. None in terms of the ones I told you about in the beginning, that there were abdominal pains, vomiting, diarrhea. He doesn’t have any symptoms like that at all.… before taking the medication, very often. If, if anything, it’s was like – he had to stay overnight at the hospital every week. The symptoms occurred almost every week.			
Facial edema: Facial edema physician assessment^b^			
2.0 (1.0–4.0) 10 (100.0)	1.0 (1.0–2.0)^c^ 10 (1001010.0)^d^	–1.0 (–3.0 to 0.0)^c^ 10 (1001010.0)^d^	Improve: 8 (80.0)^c^ No change: 2 (20.0)
Quote: PATIENT: Edema disappeared as well; the swelling I had in the face disappeared. For example, my edema was grade two-three, or maybe grade four before I was enrolled in the treatment if I am not wrong. Therefore, there is a significant regression in that. I mean, I received the medication, and almost after 1 week, for example, it was around grade one, maybe even zero, in fact there was no edema at all…. All of them, all symptoms disappeared.			
Diarrhea: PedsQL™ GI symptoms diarrhea subscale^a^			
69.65 (14.3–100.0) 10 (100.0)	100.0 (85.7–100.0) 10 (100.0)	30.35 (0.0–85.7) 10 (100.0)	Improve: 8 (80.0) No change: 2 (20.0)
Quote: INTERVIEWER: How often did she get diarrhea? CAREGIVER: As I said back then, I used to change her diaper 10 times per day but there was such a smell. However, it stopped now. There is no diarrhea…. INTERVIEWER: Don’t delay her medicine. When was the last time she had diarrhea? CAREGIVER: It has been quite some time, she hasn’t had it for a few months now.			
Peripheral edema: Peripheral edema physician assessment^b^			
2.5 (1.0–4.0) 10 (100.0)	1.0 (1.0–1.0)^c^ 10 (1001010.0)^d^	–1.0 (–3.0 to -0.0)^c^ 1010 (1090.0)^d^	Improve: 9 (90.0)^c^ No change: 1 (10.0)
Quote: INTERVIEWER: Alright, does she have it on her feet? Can you walk comfortably? PATIENT: [SPEAKING ARABIC] Yes, I can even run now. Me and my sister, we sometimes race. INTERVIEWER: Well done. Who comes first? PATIENT: [SPEAKING ARABIC] Me.… PATIENT: [SPEAKING ARABIC] No. Now that the swelling in my abdomen is gone, there is nothing left.			
Nausea and vomiting: PedsQL™ GI Symptom nausea and vomiting subscale^a^			
62.6 (0.0–100.0) 10 (100.0)	100.0 (93.8–100.0) 10 (100.0)	37.45 (0.0–100.0) 10 (100.0)	Improve: 8 (80.0) No change: 2 (20.0)
Quote: INTERVIEWER: Nausea. Now let me ask you, let’s come back to the present. The nausea, possibility of vomiting. When did it last happen? CAREGIVER: Um, wait – when did we start the trial? What month? INTERVIEWER: You can think about it. It can be approximate, mom. CAREGIVER: Before actually entering the trial – INTERVIEWER: Uh, what month, do you remember? CAREGIVER: Because it was a different week, the week before entering the trial, he still had symptoms – nausea, vomiting, stomach pain. INTERVIEWER: It was the same period as you said before?… INTERVIEWER: Ah, and vomiting, that has occurred, right? When was the last time? CAREGIVER: Well, like, around October and November, too. INTERVIEWER: Oh, okay. And stomach pain, abdominal pain? CAREGIVER: Also October and November.…. CAREGIVER: Since taking the medication, since joining the study, he hasn’t had any symptoms at all.			

GI, gastrointestinal; PedsQL™, Pediatric Quality of Life

^a^PedsQL™ subscales range from 0 to 100, where lower scores indicate worse overall health outcomes

^b^Edema scores are based on a five-point Likert scale (0–4), where a score of 0 represents “no symptoms” and 4 represents “very severe.” Higher scores indicate worse edema

^c^Data from week 20 used to calculate frequency of participants achieving response

^d^Scores were missing at week 24 for one participant, therefore week 20 score was used

### Resolution of MBS for each patient

Table 6 presents quotes from the interviews and patient-level COA scores associated with MBS from screening and week 24. These results demonstrate that, based on both the data from the interviews and the COA scores, all patients’ MBS improved or resolved following treatment. For nine patients (90.0%), data from the interviews and the COA scores demonstrate complete resolution of their MBS (i.e. a score of 100 on the PedsQL™ Pain and Hurt subscale and GIS rated as “absent”). For one patient, the caregiver reported during the interview that the MBS (abdominal pain) had resolved, indicating on the GIS that the child’s symptoms were “absent”; this patient’s PedsQL™ Pain and Hurt subscale score improved from 0 to 91.7, a nearly perfect score.

**Table 6:** Change in MBS over the clinical trial as assessed by the PedsQL™ GI Stomach Pain and Hurt subscale for abdominal pain, facial edema physician assessment, global assessments, and participant interview quotes

Pre-treatment information for MBS		Post-treatment information for MBS		Post-treatment information about change in MBS	
Participant #1 (abdominal pain) – response: resolution					
Baseline score on the PedsQL™ Pain and Hurt subscale^a^	Baseline GIS score (overall symptoms)^b^	Week 24 score on the PedsQL™ Pain and Hurt subscale^a^	Week 24 GIS score (overall symptoms)^b^	Change score PedsQL™ Pain and Hurt subscale^a^ (week 24–baseline)	GIC (overall symptoms)^b^
54.2	3 (severe)	100.0	0 absent (no symptoms)	45.8	3 (much better)
Screening interview symptom information		Exit interview symptom information		Exit interview change information	
PATIENT: I started having frequent stomach pain.… It aches often … and cramps.… It really hurts and hurts a lot.… A twisting pain.… Well, every time I eat the wrong type of food or eat food that the intestines can’t digest.… Most of the time it’s every other week.… It depends. Sometimes it’s short and goes away quickly – a few hours – but sometimes half a day or even a day. INTERVIEWER: … how bad is it? PATIENT: Very bad.		INTERVIEWER: Then after taking the drug, did you have that? CAREGIVER: After taking the drug, there were no more symptoms.… INTERVIEWER: When was the last time? PATIENT: About the end of the year, around October, November, thereabouts.… Sometimes I couldn’t run. If I was having a stomachache, I had to walk slowly. If I ran, it would make the pain worse. INTERVIEWER: Ah, and how did that change after starting to take the drug? PATIENT: I could run normally.		INTERVIEWER: So now, after taking the drug treatment, do we still have any symptoms? CAREGIVER: None; we can live normally.… INTERVIEWER: Ah. Before taking the drug, how severe was it? PATIENT: Ten. INTERVIEWER: Then after taking the drug? PATIENT: Zero. I had to wake up at night with stomach pain, throwing up, vomiting. INTERVIEWER: And was there a change? PATIENT: A change. INTERVIEWER: Do you mean after taking the drug? PATIENT: Uh, there was nothing. I bathed and slept comfortably.	
Participant #2 (abdominal pain) – response: resolution					
Baseline score on the PedsQL™ Pain and Hurt subscale^a^	Pre-treatment GIS score (overall symptoms)^b^	Week 24 score on the PedsQL™ Pain and Hurt subscale^a^	Week 24 GIS score (overall symptoms)^b^	Change score PedsQL™ Pain and Hurt subscale^a^ (week 24–baseline)	GIC (overall symptoms)^b^
20.8	4 (very severe)	100.0	0 absent (no symptoms)	79.2	3 (much better)
Screening interview symptom information		Exit interview symptom information		Exit interview change information	
INTERVIEWER: And the stomach pain, does that last for a long time? When it happens? CAREGIVER: For a long time. It begins, then it hurts and hurts again. Sometimes it hurts all day and all night. Sometimes there’s pain and vomiting too. It’s not just one episode of vomiting – it’s like a vomiting marathon, like vomiting until the insides are completely empty.		CAREGIVER: No symptoms at all. None in terms of the ones I told you about in the beginning, that there were abdominal pains, vomiting, diarrhea. He doesn’t have any symptoms like that at all.		CAREGIVER: Since taking the medication, since joining the study, he hasn’t had any symptoms at all.	
Participant #3 (abdominal pain) – response: resolution					
Baseline score on the PedsQL™ Pain and Hurt subscale^a^	Pre-treatment GIS score (overall symptoms)^b^	Week 24 score on the PedsQL™ Pain and Hurt subscale^a^	Week 24 GIS score (overall symptoms)^b^	Change score PedsQL™ Pain and Hurt subscale^a^ (week 24–baseline)	GIC (overall symptoms)^b^
83.3	1 (mild)	100.0	0 absent (no symptoms)	16.7	1 (a little better)
Screening interview symptom information		Exit interview symptom information		Exit interview change information	
PATIENT: When I go out and get cold in my stomach, I immediately experience very severe stomachache.… Then I go to bed. And my stomachache disappears as well. Afterwards, I eat some soup, and it disappears.… In the winter. It also occurs a little bit in the summer. INTERVIEWER: How many times does this occur in a month in the summer for example? PATIENT: It occurs a bit less frequently, say three times. INTERVIEWER: Is it more frequent in the winter? PATIENT: Yes.… When it is very cold in winter and when I go out and when it is snowing, it occurs every day.… It lasts from five o’clock to seven o’clock.… INTERVIEWER: Okay. Okay, how often do you vomit? PATIENT: When I have stomachache.		INTERVIEWER: How did you notice it, how did it improve, your stomachache disappear, how did you notice it? PATIENT: In other words, there was no stomachache, vomiting, nausea, and no visits to the hospital when they administered the drug. If any, it happened two-three times.		INTERVIEWER: You started taking the medication, you went to the doctor and started taking the medication. Did it change? PATIENT: I have never had abdominal pain since I have taken the medicine. INTERVIEWER: Did not it happen at all? Not for 6 months. High five! Well done.	
Participant #4 (facial edema) – response: resolution					
Baseline score on the facial edema physician assessment^c^	Pre-treatment GIS score (overall symptoms)^b^	Week 24 score on the facial edema physician assessment^c^	Week 24 GIS score (overall symptoms)^b^	Change score facial edema physician assessment^c^ (week 24–baseline)	GIC (overall symptoms)^b^
4 (severe edema)	1 (mild)	1 (no edema)	0 absent (no symptoms)	–3	3 (much better)
Screening interview symptom information		Exit interview symptom information		Exit interview change information	
CAREGIVER: It was 2 years ago in the morning and when I woke up, I checked him; he had swelling on his feet and his eyelids.… By no means. I got an understanding just by looking at the edema on his feet and eyelids. I mean, why was this child swollen like this, his undereyes, eyelids, feet.… INTERVIEWER: Edema? Tell me where it used to be. CAREGIVER: The same eyelids, under the eyes and on his feet, but they were getting worse. INTERVIEWER: I mean, how soon does it start; for example, it starts one day and then? CAREGIVER: For example, when I got up one morning, if he had the same edema, I would immediately take him to the hospital.		INTERVIEWER: Alright, if I particularly ask about the swelling in the face, did you follow it up? How did you understand s/he did not experience it? Were you checking her/his face? CAREGIVER: Sure, I was continuously following it up, and I understood that it did not occur. … INTERVIEWER: Something like I used to see this most before the drug that comes from the United States of America was administered, the first thing I observed to disappear was this, I looked and I understood from here, etc. CAREGIVER: Swelling disappeared first.		CAREGIVER: The bad symptoms disappeared. INTERVIEWER: For example, what did get back in order? Please tell me step by step. CAREGIVER: What do you mean? INTERVIEWER: Please tell me step by step, what did get well; I mean how did s/he have relief? CAREGIVER: Swelling disappeared; nausea, abdominal pain, diarrhea; all of these disappeared. These were the complaints of her/his disease on their own, but these disappeared.	
Participant #5 (abdominal pain) – response: resolution					
Baseline score on the PedsQL™ Pain and Hurt subscale^a^	Pre-treatment GIS score (overall symptoms)^b^	Week 24 score on the PedsQL™ Pain and Hurt subscale^a^	Week 24 GIS score (overall symptoms)^b^	Change score PedsQL™ Pain and Hurt subscale^a^ (week 24–baseline)	GIC (overall symptoms)^b^
62.5	2 (moderate)	100.0	0 absent (no symptoms)	37.5	3 (much better)
Screening interview symptom information		Exit interview symptom information		Exit interview change information	
INTERVIEWER: Alright, does it sometimes occur during the day or can it occur at a certain period of time, or can it occur any time? PATIENT: It can happen any time.… PATIENT: I feel sad when I have a stomachache. INTERVIEWER: You are sad, why are you sad? PATIENT: Because the pain is terrible. When I have a terrible pain, I have nausea as well.		PATIENT: I do not have any stomachache at the moment. INTERVIEWER: Really? PATIENT: Yes.… PATIENT: I used to have a headache, stomachache, nausea, all of these complaints in the past. Now I don’t have any of these.		INTERVIEWER: Okay, how did you notice that this stomachache changed? Did it change? PATIENT: I do not have any stomachache at the moment. INTERVIEWER: Really? PATIENT: Yes.	
Participant #6 (abdominal pain) – response: resolution					
Baseline score on the PedsQL™ Pain and Hurt subscale^a^	Pre-treatment GIS score (overall symptoms)^b^	Week 24 score on the PedsQL™ Pain and Hurt subscale^a^	Week 24 GIS score (overall symptoms)^b^	Change score PedsQL™ Pain and Hurt subscale^a^ (week 24–baseline)	GIC (overall symptoms)^b^
83.3	1 (mild)	100.0	0 absent (no symptoms)	16.7	3 (much better)
Screening interview symptom information		Exit interview symptom information		Exit interview change information	
INTERVIEWER: Exactly where does she feel the pain? CAREGIVER: Bottom part of the tummy, from the right to the left, underneath the belly button. So it starts from the level beneath the abdomen, starting from here on the right, she feels pain till the left, completely.		INTERVIEWER: Were you able to sleep when she had this CD55? PATIENT: No. INTERVIEWER: What happened? PATIENT: I mean, I would have a stomachache and I could not sleep when I had a stomachache. INTERVIEWER: Did you wake up from your sleep? PATIENT: Yes. INTERVIEWER: How about now? PATIENT: Now I can sleep until 10:00 a.m., 11:00 a.m.… CAREGIVER: She is saying the same thing. So, it has come from the worst to the best. INTERVIEWER: You had said that stomachache was 10 and from 10 to 0. CAREGIVER: That was really bad anyway, thank God it is very good now.		INTERVIEWER: What about the stomachache? CAREGIVER: Yes, it has disappeared. It is gone.… INTERVIEWER: Then diarrhea was affected first and this was followed by stomachache.	
Participant #7 (abdominal pain) – response: resolution					
Baseline score on the PedsQL™ Pain and Hurt subscale^a^	Pre-treatment GIS score (overall symptoms)^b^	Week 24 score on the PedsQL™ Pain and Hurt subscale^a^	Week 24 GIS score (overall symptoms)^b^	Change score PedsQL™ Pain and Hurt subscale^a^ (week 24–baseline)	GIC (overall symptoms)^b^
58.3	1 (mild)	100.0	0 absent (no symptoms)	41.7	3 (much better)
Screening interview symptom information		Exit interview symptom information		Exit interview change information	
INTERVIEWER: For example, were they able to understand if the child did not say that her/his stomach hurt? CAREGIVER: Yes. INTERVIEWER: How? CAREGIVER: Sometimes s/he pointed. INTERVIEWER: To where? CAREGIVER: The abdominal area. INTERVIEWER: To the part where the belly button is? CAREGIVER: Yes. S/he laughed sometimes, then did nothing when it was bad, s/he lay like that. Sometimes something like an egg was floating around in her/his stomach. INTERVIEWER: What does that mean? CAREGIVER: I mean, there is a swollen part like an egg in your intestines.… INTERVIEWER: How do they know that? CAREGIVER: In other words, they sensed that something was moving in her/his stomach. INTERVIEWER: They saw it. CAREGIVER: Yes. S/he had relief when s/he pooed. INTERVIEWER: Did s/he have gas discharge? CAREGIVER: No, not gas. INTERVIEWER: How come s/he had relief when s/he pooed? CAREGIVER:.… Something was floating, and s/he finally had relief when s/he discharged it, pooed in the restroom.		INTERVIEWER: Alright, they observed this. Alright, they said the stomachache disappeared. Do they observe that the abdominal pain is gone, or does the child tell? CAREGIVER: Both. We observe it and s/he tells it. INTERVIEWER: Okay. For example, as far as I know, s/he used to have injections. Did they ask the child afterwards? CAREGIVER: Of course we asked and s/he answered. INTERVIEWER: Yes, but, for example, I mean, s/he is not twisted in pain but s/he has mild pain. CAREGIVER: No. INTERVIEWER: Alright, this is interesting, it dropped down. Do they think that it is a significant change? CAREGIVER: Yes, it changed completely.		INTERVIEWER: Alright, they observed this. Alright, they said the stomachache disappeared. Do they observe that the abdominal pain is gone, or does the child tell? CAREGIVER: Both. We observe it and s/he tells it.	
Participant #8 (abdominal pain) – response: resolution					
Baseline score on the PedsQL™ Pain and Hurt subscale^a^	Pre-treatment GIS score (overall symptoms)^b^	Week 24 score on the PedsQL™ Pain and Hurt subscale^a^	Week 24 GIS score (overall symptoms)^b^	Change score PedsQL™ Pain and Hurt subscale^a^ (week 24–baseline)	GIC (overall symptoms)^b^
8.3	2 (moderate)	100.0	0 absent (no symptoms)	91.7	3 (much better)
Screening interview symptom information		Exit interview symptom information		Exit interview change information	
INTERVIEWER: Could you tell me how you feel now due to the stomachache? Are there any other words you can use for describing it? PATIENT: If my abdominal pain is severe, I even cannot get up. I literally cannot get up if I do not have someone next to me to take my arm and help me in getting up. I started experiencing this more often for a few years, 3, even 4 years. Previously, I was able to handle it somehow when I had pain, good or bad, but for a couple of years at least, the situation is not like that. It started to be worse and make me feel more exhausted. After I have abdominal pain, I feel drowsy. That day is literally wasted. It is very rare that I can continue my day. Like that.		PATIENT: The first day I received the medicine, I presented to the hospital with pain. I got up sick and went to the hospital to receive the medicine. I went there and it was effective a short time after I received it, almost an hour, and I understood that a mild cramp was there in my abdomen and that severe pain disappeared. I mean, it immediately disappeared; all of my pain disappeared.		INTERVIEWER: Alright; did the abdominal pain disappear? You wanted that one most to disappear. PATIENT: Yes, that one directly disappeared.	
Participant #9 (abdominal pain) – response: improvement					
Baseline score on the PedsQL™ Pain and Hurt subscale^a^	Pre-treatment GIS score (overall symptoms)^b^	Week 24 score on the PedsQL™ Pain and Hurt subscale^a^	Week 24 GIS score (overall symptoms)^b^	Change score PedsQL™ Pain and hurt subscale^a^ (week 24–baseline)	GIC (overall symptoms)^b^
0	1 (mild)	91.7	0 absent (no symptoms)	91.7	3 (much better)
Screening interview symptom information		Exit interview symptom information		Exit interview change information	
INTERVIEWER: Alright, how about that thing? You said that she would also have abdominal pain, that you saw it like that. How did you understand that? CAREGIVER: Ms. her stomach would feel hard like a stone. But she would have diarrhea and she would squirm. But the diarrhea, how can I say this, it would get really bad.… CAREGIVER: Once these four [symptoms] occurred, she would get abdominal pain and diarrhea immediately afterwards. INTERVIEWER: Okay, how would you observe that your daughter was feeling bad? CAREGIVER: She would bend herself and then she would be tense. You know, babies bend, pull themselves backwards and then squirm when they have pain, exactly like that. That is how I understood it.		INTERVIEWER: Okay, do you think this change or the fact that her abdominal pain is resolved, is meaningful or important to your child? CAREGIVER: Of course, Ms., not only for her health but also for her development. I could not tell her anything in the past, but now as her abdominal pain has disappeared, her movements have become faster compared to that thing. Her movements have become faster and she shows more effort now. INTERVIEWER: Great. This is really good news. Alright, how else did the resolution of this abdominal pain affect her daily life? CAREGIVER: Ms., when she got abdominal pain, she would always squirm on the ground but when it was resolved, how can I say this, she tries to enter all kinds of holes, she moves more comfortably. She goes through all kinds of places.		CAREGIVER: The swelling in her eyes disappeared. Her abdominal pain, fever, and diarrhea symptoms were resolved completely. I mean, she had a lot of improvement.	
Participant #10 (Abdominal pain) – response: resolution					
Baseline score on the PedsQL™ Pain and Hurt subscale^a^	Pre-treatment GIS score (overall symptoms)^b^	Week 24 score on the PedsQL™ Pain and Hurt subscale^a^	Week 24 GIS score (overall symptoms)^b^	Change score PedsQL™ Pain and Hurt subscale^a^ (week 24–baseline)	GIC (overall symptoms)^b^
41.7	3 (severe)	100	0 absent (no symptoms)	58.3	3 (much better)
Screening interview symptom information		Exit interview symptom information		Exit interview change information	
INTERVIEWER: OK. And what was the main reason why you went to the doctor? PATIENT: Lots of diarrhea, vomiting and, finally, I started having stomachaches. I got very dehydrated and they took me (to the hospital) and hooked me to a saline drip, painkillers and sent me back home. At [REDACTED] all they did was give me a saline drip and painkillers, and they would send me back home. They didn’t prescribe any treatment.… When my stomach aches, I get stomachache and vomiting, but sometimes without diarrhea.… INTERVIEWER: Describe your stomachache in more detail. What does it feel like? PATIENT: I feel tightness; my guts are straining, something like that; it starts to cramp and when this happens I want to throw everything up, and sometimes throwing up helps with the pain. I throw up some yellow fluid.		INTERVIEWER: And when was the last time you had a stomachache? Do you remember? PATIENT: A day before I received the medicine. INTERVIEWER: And after taking the medicine, were you ever in pain? PATIENT: It no longer hurt.… INTERVIEWER: You haven’t had any symptoms of diarrhea since you started taking the medicine? PATIENT: Since I started taking the medicine; I haven’t had any diarrhea, vomiting or stomachaches.… Now I feel normal; I am no longer in pain; I can do whatever I want.… PATIENT: None. No symptoms.… INTERVIEWER: Can you describe the different symptoms? The stomachache was the worst. And how did that pain go away? PATIENT: When they gave me the medicine.		INTERVIEWER: And when was the last time you had a stomachache? Do you remember? PATIENT: A day before I received the medicine. INTERVIEWER: And after taking the medicine, were you ever in pain? PATIENT: It no longer hurt.	

GI, gastrointestinal; GIC, global impression of change; GIS, global impression of change; MBS, most bothersome sign or symptom; PedsQL™, Pediatric Quality of Life

^a^PedsQL™ subscales range from 0 to 100, where lower scores indicate worse overall health outcomes

^b^GIS/GIC reported by patient or caregiver

^c^Edema scores are based on a five-point Likert scale (0–4), where a score of 0 represents “no symptoms” and 4 represents “very severe.” Higher scores indicate worse edema

## Discussion

CHAPLE disease is an ultra-rare condition [1, 4] for which pozelimab treatment was recently approved by the FDA [6]. Interviews with patients and/or caregivers were conducted as part of the pozelimab clinical trial to further elucidate the patient experience by contextualizing the change in COA scores and meaningfulness of the change, including signs and symptoms not explicitly measured in the trial. This is critical for drug evaluation because the FDA standard for a clinically positive effect of a new drug, i.e. efficacy, is whether it improves how a patient feels, functions, or survives [21]. Interviews completed at the screening visit confirmed that the signs and symptoms identified as core through a review of the literature [1, 3, 7] and discussion with treating clinicians (i.e. abdominal pain, facial and peripheral edema, diarrhea, nausea, and vomiting) were indeed central to the disease experience. Further, abdominal pain was identified by most patients as the MBS that they wanted to see improve with treatment. The week 24 interviews demonstrated that all but one sign improved following 24 weeks of pozelimab treatment, no sign/symptom worsened, and that all core signs/symptoms had completely resolved.

The COA measurement strategy for the trial was based on the concepts identified in the literature and discussed in meetings with treating clinicians, and there was therefore a risk that these concepts would not be confirmed during the within-trial interviews. However, concepts covered by the COAs included in the trial were identified as core signs and symptoms during the screening interviews. Consequently, the COA measurement strategy comprehensively captured CHAPLE disease. Further, an added benefit of the data gathered from the interviews was the opportunity to assess change in signs and symptoms that were not targeted for assessment via COAs, which helped to further contextualize the pozelimab treatment effects on the disease experience holistically. Specifically, the interview data contextualized meaningful within-person change on COA scores, which would have not been possible to evaluate quantitively given the small sample size.

The mixed method analyses specific to each participants’ MBS also demonstrated resolution of the single sign/symptom that was noted before treatment as being the most bothersome. While the use of MBS has been reported in the literature [12, 16, 22, 23] and in an FDA regulatory guidance document for migraine [17], to our knowledge this is the first use of the MBS approach in an ultra-rare disease trial. Nearly all participants experienced all six core signs/symptoms of disease before starting pozelimab and nine participants had abdominal pain as their MBS, suggestive of a homogenous experience of disease. Given the paucity of information on the patient experience during protocol development, use of the MBS approach could have been helpful in interpreting the results of the trial had the CHAPLE disease experience been more heterogenous.

Overall, interview results aligned with the changes in scores on the COAs and global assessments of CHAPLE disease activity from the perspective of the participants and treating physicians. When looking at the responses to the COAs in the clinical trial, findings are largely consistent with those from the interviews and provided further evidence of the meaningfulness of the change experienced after treatment.

The results of this study must be interpreted in the context of certain limitations. The COAs for the clinical trial were selected to overlap conceptually with the core signs and symptoms; however, there may be differences between patient- or caregiver-reported concepts and the concepts in the COAs, as well as the way that the concepts were reported and measured (e.g. different time frames/recall periods). Given the wide age range of patients, not all patients could be the primary respondent and questionnaires appropriate for caregiver report of symptoms were selected. During the qualitative interviews, however, caregivers were asked to explain how they were aware of the symptom and how they gauged severity. Caregivers reported both verbal and nonverbal ways the child communicated their symptoms and the severity of their symptoms [15]. Depending on age, global assessments were completed by either by caregivers or by patients (depending on age), as well as clinicians, and while caregivers/patients and clinicians rating of baseline assessments differed for some patients, all scores at week 24 demonstrated the resolution of signs/symptoms, including patients who had scores of “mild” before treatment. It should be noted that all patients enrolled in the clinical trial had active disease and hypoalbuminemia [14]. Finally, the trial design (single-arm) and sample size might be viewed as a limitation; however, it was considered adequate given the overall prevalence of CHAPLE disease (< 100 patients worldwide) and the life-threatening nature of the condition [14].

## Conclusion

Findings from the within-trial interviews align with the change in COA scores and the patient or caregiver and clinical global assessments of CHAPLE disease activity. The mixed-methods approach allowed for a more comprehensive assessment of the patient experience in a trial evaluating a treatment for an ultra-rare and newly discovered disease by assessing the efficacy of treatment for signs and symptoms in the trial, including those that were not measured by COAs. Together, the results from the interviews and COAs comprehensively contextualized the patient experience and demonstrated the efficacy of pozelimab on the signs and symptoms of CHAPLE disease, as well as the transformative impact treatment had on the lives of patients. This approach may be especially useful for meeting the FDA requirements for drug efficacy, especially for rare and severe diseases.

### Electronic supplementary material

Below is the link to the electronic supplementary material.

Supplementary Material 1

## Data Availability

Qualified researchers may request access to study documents (including the clinical study report, study protocol with any amendments, blank case report form, and statistical analysis plan) that support the methods and findings reported in this manuscript. Individual anonymized participant data will be considered for sharing once the product and indication has been approved by major health authorities (e.g. FDA, European Medicines Agency, Pharmaceuticals and Medical Devices Agency, etc.), if there is legal authority to share the data and there is not a reasonable likelihood of participant re-identification. Requests should be submitted to https://vivli.org/.

## Abbreviations

COA: Clinical outcome assessment
CHAPLE: CD55 deficiency with hyper-activation of complement, angiopathic thrombosis, and protein- losing enteropathy
FDA: Food and Drug Administration
GIC: Global impression of change
GIS: Global impression of severity
IV: Intravenous
MBS: Most bothersome sign or symptom
PedsQL™: Pediatric Quality of Life Inventory
SC: Subcutaneous
