# Supplementary material for: Pozelimab for CHAPLE disease: results from in-trial interviews and clinical outcome assessments
Source: Orphanet J Rare Dis. 2024 Aug 8;19:290. doi: 10.1186/s13023-024-03277-9 (PMC11308501; doi:10.1186/s13023-024-03277-9)
Supplement: Supplementary file 1 — Supplementary Material 1 [file 13023_2024_3277_MOESM1_ESM.docx]

# Additional file 1: Information on clinical outcome assessments for the core signs and symptoms

The PedsQL™ gastrointestinal pain and hurt, diarrhea, and nausea/vomiting subscales ask the patient (if aged ≥12 years) or caregiver (if the patient is aged <12 years) to rate how much of a problem each item concept has been over the past 7 days on a five-point response scale, ranging from never (0) to almost always (4). Items are reverse-scored and transformed to a 0––100 scale so that higher scores indicate less frequency of problems with gastrointestinal signs and symptoms. Scores were calculated for each subscale.

The facial and peripheral edema assessments were constructed specifically for this trial, and were completed by the treating physician. For facial edema, the physician examined the patient’s face and rated the overall severity of facial edema on a five-point rating scale: (1) non edema; (2) mild edema (slight puffiness around the eyes with some flattening of superficial creases); (3) moderate edema (definite periorbital swelling with some flattening of deep creases with or without puffiness of the cheeks); (4) severe edema (marked periorbital swelling with loss of deep periorbital creases and definite swelling of the cheeks); and (5) very severe edema (marked swelling of the entire face, eyes can only open to slit-like apertures). Similarly, for peripheral edema, the physician performed a general inspection and palpation of all four limbs and rated the overall severity of peripheral edema on five-point rating scale: (1) no edema, (2) mild edema (slight pitting, no visible change in the shape of the extremity); (3) moderate edema (definite pitting, slight change in the shape of the extremity); (4) severe edema (deep pitting, swollen extremity); and (5) very severe edema (very deep pitting, very swollen, distorted extremity).
